# Prediction and Prevention of Type 1 Diabetes

**DOI:** 10.3389/fendo.2020.00248

**Published:** 2020-06-02

**Authors:** Marina Primavera, Cosimo Giannini, Francesco Chiarelli

**Affiliations:** Department of Pediatrics, University of Chieti, Chieti, Italy

**Keywords:** type 1 diabetes, children, prediction, primary prevention, secondary prevention, tertiary prevention

## Abstract

Type 1 Diabetes (T1D) is one of the most common chronic autoimmune diseases in children. The disease is characterized by the destruction of beta cells, leading to hyperglycemia, and to a lifelong insulin-dependent state. Although several studies in the last decades have added relevant insights, the complex pathogenesis of the disease is not yet completely understood. Recent studies have been focused on several factors, including family history and genetic predisposition (HLA and non-HLA genes) as well as environmental and metabolic biomarkers, with the aim of predicting the development and progression of T1D. Once a child becomes symptomatic, beta cell mass has already reached a critical threshold (usually a residual of 20–30% of normal amounts), thus representing only the very late phase of the disease. In particular, this final stage follows two preceding asymptomatic stages, which have been precisely identified. In view of the long natural history and complex pathogenesis of the disease, many strategies may be proposed for primary, secondary, and tertiary prevention. Strategies of primary prevention aim to prevent the onset of autoimmunity against beta cells in asymptomatic individuals at high risk for T1D. In addition, the availability of novel humoral and metabolic biomarkers that are able to characterize subjects at high risk of progression, have stimulated several studies on secondary and tertiary prevention, aimed to preserve residual beta cell destruction and/or to prolong the remission phase after the onset of T1D. This review focuses on the major current knowledge on prediction and prevention of T1D in children.

## Introduction

Type 1 diabetes (T1D) is a chronic autoimmune disease characterized by pancreatic beta cell destruction in which genetic susceptibility combined with environmental factors, mostly in early life, plays a crucial role. Several studies have been focusing on the identification of individuals at risk for T1D, early in the natural history of the disease, using prediction models in which the genetic factors are considered to be important for their time-independence in all subjects. These results have offered the possibility of identifying people at risk and to follow them during the years, in order to try to prevent or revert the progression of T1D. Nevertheless, genetic factors do not provide a sufficient explanation regarding the development of the disease. In the last decade, the Eisenbarth model has tried to explain the progression of T1D (1), suggesting three main stages in the natural history of T1D. The first stage is featured by the presence of autoantibodies (at least two islet autoantibodies) with normal blood glucose levels and no symptoms (stage 1, or the “asymptomatic phase”) (2). In genetically predisposed individuals, environmental factors could act as a trigger of T-cell and humoral autoimmune responses against beta cells (3). Stage 2 is defined by the positivity of two or more autoantibodies with alterations of glucose metabolism not diagnostic for diabetes still in absence of clinical symptoms (“early metabolic alterations with asymptomatic state”). “Clinical diabetes,” or stage 3, is characterized by the onset of clinical manifestations (Table 1) (4). The duration of each phase and the risk of progression from one stage to the other are not completely known. At the moment, one relevant focus is to characterize each phase of this complex disease in order to predict and prevent T1D, which is the dream as well as the most challenging obstacle for clinicians and scientists. This review has the aim to describe the most recent knowledges on the main and recent strategies of prediction and prevention of T1D.

**Table 1:** Staging of Type 1 Diabetes according to JDRF, the Endocrine Society, and the American Diabetes Association (4).

**Stage 1**	**Stage 2**	**Stage 3**
Beta cell autoimmunity	Beta cell autoimmunity	Beta cell autoimmunity
Normoglycemia	Dysglycemia	Dysglycemia
Presymptomatic	Presymptomatic	Symptomatic

### Predictors of Risk for T1D

Ongoing research on T1D has produced abundant data evaluating potential predictive factors associated with the risk of beta cell destruction. Although several factors have been proposed, the genetic, infective, dietary, and humoral factors are the most relevant. More importantly, due the multifactorial nature of the disease, these factors might be considered not individually but as being on a spectrum and interactive factors that if combined might strongly enhance the risk of developing the disease. Therefore, the complete characterization of each of these components might be of relevance in order to properly define the risk of T1D development.

### Genetic Factors

In T1D, a clear pattern of inheritance is lacking; nevertheless, many studies have reported that genetic predisposition might explain up to 50% of the risk (5). Relatives of T1D patients have higher risk of developing T1D (about 15–20 times, since the risk is about 0.4% among the general population) (6, 7). The concordance rate for T1D is, respectively, 25–50% in identical twins and 6–7% in dizygotic twins and siblings (7, 8). The human leukocyte antigen (HLA) complex plays a critical role in the pathogenesis of T1D, representing a substantial component of the genetic risk (about 50%). The HLA region on chromosome 6p21 encodes class-I, class-II, and class-III genes. The telomeric boundary of the locus comprises the class-I genes, including HLA-A, HLA-B, and HLA-C, whereas the centromeric boundary comprises the class-II genes, including HLA-DP, HLA-DQ, and HLA-DR. Class III is located in the middle part of the HLA region (9). Combinations of specific alleles of HLA class II strongly influence the risk of T1D. For example, the combination of HLA-DRB1^*^04 with DQA1^*^03:01-DQB1^*^03:02 (known as DR4-DQ8) increases the risk of developing T1D, while HLA DRB1^*^04 combined with DQA1^*^03-DQB1^*^03:01 does not (10, 11). The highest risk of T1D is linked not only to DR4-DQ8 haplotype, but also to another class-II haplotype known as DR3-DQ2 (DRB1^*^03:01-DQA1^*^05:01-DQB1^*^02:01) (2). HLA is involved in the immune process of antigen presentation; therefore, it is clear how this gene region can influence both etiology and pathogenesis of T1D, and this is confirmed by the sequence of appearance of islet autoantibodies. Insulin autoantibodies (IAA) appear in children up to 6 years of age with DR4-DQ8 haplotype, while GAD65 autoantibodies first appear in carriers of DR3-DQ2 (12). If the haplotype of HLA influences the appearance of the first autoantibody, no similar associations are reported for the appearance of subsequent autoantibodies (13). Some haplotypes could be protective factors for the development of T1D for example, DQB1^*^06:02-DRB1^*^15:01-DQA1^*^01:02 (also known as DR2) is detected in ~20% of the individuals, but in only 1% of patients with T1D (14). HLA class I is expressed in all nucleated cells, and it is also involved in the antigen-presenting process to lymphocytes. However, the risk for T1D in patients with HLA class-I haplotypes is relatively low compared to those with HLA-DR and HLA-DQ (15). In addition, it is important to underline that <10% of individuals with HLA-conferred susceptibility develop T1D (16). Therefore, new genes probably need to be characterized to better define the risk of the disease. In fact, to date our knowledge on HLA haplotypes does not completely define the genetic risk of the disease, suggesting the direct effects of other genes (17, 18). Thus, non-HLA genes have been described as likewise playing a pivotal role in the pathogenesis of T1D as with other autoimmune diseases. Amon them, particularly the genes encoding, respectively, for pre-proinsulin (INS), or protein tyrosine phosphatase (PTPN22) or IL-2 receptor subunit alpha (IL2RA) are largely described (19). Other genes have been identified by genome wide association study (GWAS); among these, the 6q22.23 chromosomal region encoding protein tyrosine phosphatase receptor kappa (PTPRK) and thymocyte expressed molecule involved in selection (THEMIS) are well-studied for their critical role in thymic T cell development (20). In addition, genetic scores were proposed in recent years in order to evaluate the combined effects of different genes on the risk of T1D. Among them, Type 1 Diabetes Genetic Risk Score (T1D GRS) has been validated to predict progression of islet autoimmunity and development of T1D in at-risk individuals. Oram et al. (21) have validated a T1D GRS that incorporates HLA and non-HLA genes T1D-associated single nucleotide polymorphisms (SNPs) and that also discriminates T1D from Type 2 diabetes (T2D), monogenic diabetes, and controls (22). Redondo et al. have tested the prognostic utility of T1D GRS to differentiate rates of progression of autoimmunity against beta cells and development of clinical T1D in autoantibody-positive relatives of patients with T1D (23). GRS can predict more than 10% of risk for pre-symptomatic T1D in children without afflicted first-degree relatives (24).

### Infections

Childhood infections are surely among the most widely studied factors. The role of viral infections in the pathogenesis of T1D is supported by epidemiological, serological, and histological studies. Two main hypotheses have been proposed: the hygiene hypothesis and the triggering hypothesis. It has been speculated that infections in early childhood may be a protection against T1D as described in explanations of childhood allergy. On the other hand, specific or combined infections might cause T1D by destroying pancreatic beta cells (25). Among viruses, enteroviruses are the most commonly studied. The Diabetes Prediction and Prevention (DIPP) study demonstrated a relationship between the enteroviruses infection and the appearance of first autoantibody (26, 27); in particular, early serological studies suggested coxsackie B viruses (CBVs), especially the CBV4 serotype, may be linked to T1D (28, 29). In contrast, the role of rubella infection is controversial, because an atypical form of T1D without islet autoimmunity is described in congenital rubella syndrome. It is interesting to observe the correlation in young children between respiratory infections and the increased risk of islet autoimmunity described in The Environmental Determinants of Diabetes in the Young (TEDDY) study. The incidence of islet autoimmunity has a peak between 6 and 9 months, followed by a decline; the same trend is described for respiratory infections episodes (30). Although these results add relevant information, further studies are needed in order to properly define the role of viruses and infections in the risk of T1D in children and adolescents.

### Diet

The role of diet in T1D history is not fully understood, and the results are still conflicting. Cow's milk proteins have been proposed as triggers of an autoimmune response in hosts at genetic risk, leading to pancreatic beta cell destruction (31–35). Studies in animals have suggested that bovine serum albumin (BSA) is the milk protein responsible of the development of diabetes (31). Karjalainen et al. have studied the serum of 142 Finnish children with newly diagnosed insulin-dependent diabetes mellitus, 79 healthy children and 300 adult blood donors (32); all diabetic patients had increased serum concentrations of anti-bovine serum albumin (BSA) antibodies at the beginning of the disease. Anti-BSA antibodies were predominantly IgG and react against an albumin peptide containing 17 amino acids (ABBOS) (32). This epitope could cross-react with a beta cell surface protein 69 kd in size (p69) inducible by interferon gamma representing the target antigen for milk-induced beta cell-specific immunity. The Diabetes Autoimmunity Study in the Young (DAISY) has shown that only in low-/moderate-risk HLA-DR individuals, was the intake of cow's milk protein associated with a higher risk of developing beta cell autoimmunity, at variance of children at high risk (33). These results have been confirmed by the Trial to Reduce Insulin-Dependent Diabetes Mellitus in the Genetically at Risk (TRIGR), since no difference between the ingestion of cow's milk and the ingestion of hydrolyzed formula was found (34, 35).

Conflicting results have been also described on the use of vitamin D. Several studies demonstrate the beneficial effect of vitamin D supplementation against some autoimmune diseases (36). It has been demonstrated that all cells of the immune system have vitamin D receptors, and thus they could be regulated by calcitriol (37). Vitamin D influences the innate immune system cells (dendritic cells and macropaghes) as well as the adaptive immune system cells (B and T lymphocytes). Calcitriol enhances the tolerogenic status which results in a suppression and increase of pro-inflammatory and anti-inflammatory cytokines, respectively. It also reduces the expression of MHC class I and II and costimulatory molecules (38). Regarding vitamin D and T1D, it would seem that calcitriol supplementation would reduce serum levels of antibodies and delay the progression of beta cell destruction but only in the early stages of the disease (39). This could explain the reported controversial results. A recent study shows that the integration of vitamin D with ω-3 co-supplementation and arachidonic acid reduction in the Mediterranean diet have benefits for T1D children at onset (40). On the other hand, in the Type 1 Diabetes Prediction and Prevention Study (DIPP), Mäkinen et al. compared the 25(OH)D umbilical cord serum concentration of 764 children born between 1994 and 2004 who participated in DIPP in Finland. Results reported in this study have shown that fetal vitamin D status, measured through the concentration of 25(OH)D in umbilical cord serum, is not linked to the islet autoimmunity (41). Although these results add relevant information on the risk of T1D, other components still need to be evaluated. In fact, it might be postulated that a complex combination of early-life and probably even fetal-life factors influence the development of pancreatic autoimmunity. Understanding the burden of each of these components is the way to strategically prevent one of the most demanding chronic illnesses in children.

### Serological Biomarkers

The characterization of serological biomarkers that evaluate the pancreatic autoimmunity and the beta cell dysfunction or death represents an effective way to try to outline the progression of the disease. The positivity of autoantibodies against beta cells and the combination of them are considered the main relevant strategies to predict T1D progression. There are five primary types of islet autoantibodies: autoantibodies against insulin (IAA), autoantibodies against insulinoma-associated antigen-2 (IA-2), autoantibodies against glutamic acid decarboxylase (GAD), autoantibodies against zinc-transporter 8 (ZnT8), and islet cell antibodies (ICA) (42). Although these autoantibodies could appear at any age, they rarely appear before the age of 6 months (43). The peak incidence of appearance of a first islet autoantibody is before the age of 3 years (43–45). After this age the risk of developing islet autoimmunity declines. Both the young age of seroconversion and the positivity for multiple autoantibodies are considered the major risk factors for the development of the disease. Ziegler et al. have demonstrated that the progression to clinical T1D was faster in children who had the appearance of autoantibodies against beta cells before the age of 3 years than those who were 3 years old or older (46). In addition, progression to T1D at 10-year follow-up was about 14.5% in 474 children with a single islet autoantibody, in contrast to 69.7% in 585 children with multiple islet autoantibodies. By the age of 15 years the risk of diabetes was about 0.4% in children without islet autoantibodies (46). The titers of autoantibodies also influence the risk of progression; high titer of islet cell autoantibodies of IAA and IA-2 is associated with a high risk of progression in the 5 years following the appearance of the first autoantibody. In contrast, GADA concentrations did not differ between progressors and non-progressors (47). Nevertheless, it is important to underline that the role of islet autoantibodies positivity and titers have not a clear prognostic significance because a revert to seronegativity was found up to 60% of individuals with a single autoantibody and the antibody titers may actually change (48, 49). To date, islet autoantibody remains as the gold standard for risk stratification for the development of clinically manifest T1D, although not even the positivity of multiple autoantibodies is specific for the disease.

**Figure 1:**
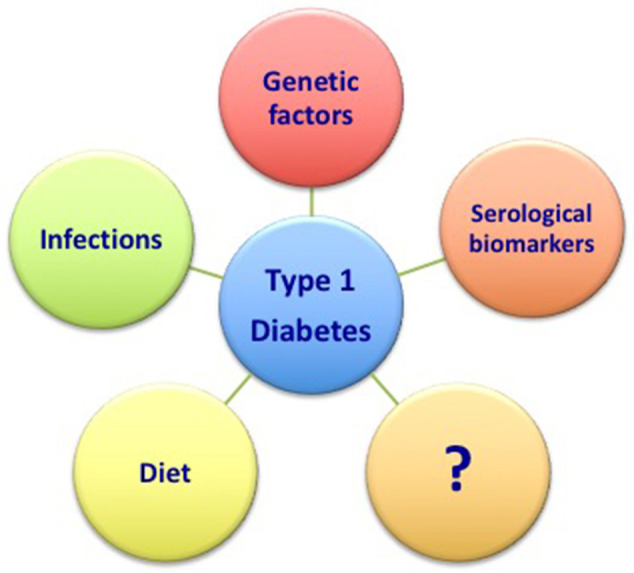
Main predictive factors associated to the risk of T1D.

Therefore, the better characterization of the main risk factors previously discussed (namely genetic factors, the role of infections, diet, and serological markers) combined with the definition of novel and still unknown factors will surely help in the future to predict the development of the disease (Figure 1). Further, ongoing researches will likely offer new perspectives in this field.

### Prevention

Prediction strategies are important to avoid the development of autoimmunity processes in subjects at risk of T1D. More importantly, they are extremely relevant to stop the natural progression of the disease. To date there are three levels of prevention: primary prevention, intended for individuals at high risk of developing T1D and aimed at preventing the autoimmunity against islet autoantigens; secondary prevention, which relates to individuals with multiple islet autoantibodies with the aim of halting autoimmunity processes and possibly avoid the clinical onset of diabetes; and, once the disease is clinically manifested, the tertiary prevention of T1D that is focused on complications of the disease, attempting to reduce or minimize these with the main goal at least of delaying their onset (50).

### Primary Prevention

Strategies for primary prevention must be started early in life, because when the earlier process of beta cell autoimmunity is initiated, the progression to T1D accelerates significantly (46). The POInT study, an investigator-initiated, randomized, placebo-controlled, double-blind, primary prevention trial has been started through a network of collaborating clinical study centers from European countries in Belgium, Germany, Poland, the United Kingdom, and Sweden. This study seeks to determine whether daily administration of oral insulin, from the age of 4–7 months until the age of 36 months to children with elevated genetic risk for T1D, reduces the incidence of beta cell autoantibodies and diabetes (51). The rationale of this study was that immunological tolerance can be achieved by the administration of antigens (52, 53). However, although the rationale of this study is very promising, complete data are not yet available; we will probably have new data in the years to come. Nevertheless, a previous study conducted between March 2, 2007 and December 21, 2015 has demonstrated that oral insulin at a dose of 7.5 mg/d, compared with a placebo, did not delay or prevent the development of T1D over 2.7 years in autoantibody-positive relatives of T1D patients (54).

Due to the potential role of infections in the pathogenesis of T1D, the opportunity to administer a vaccine against viruses associated to T1D is being explored. In particular, the Juvenile Diabetes Research Foundation (JDRF) is now funding research in this field, likely offering promising perspectives in the near future (55). Under development are not only viral vaccines, but also vaccines inducing immune tolerance to beta cell antigens (56, 57). Neoepitopes are very important because they could be an alternative antigenic target for T1D tolerogenic vaccines.

The role of gut microbiome is critical for the immune regulation, education, and maturation of the immune system in infants. Several cohorts have been studied in order to investigate the relationship between early microbiome or its perturbations with the development of islets autoantibodies. Studies are underway in order to clarify the role of intestinal bacterial diversity in inducing the risk of T1D development in children. In the TEDDY study, modest alterations of microbial composition have been found in patients with islet autoantibodies or T1D not revealing clear taxonomic differences (58, 59). However, a relevant point is that the microbiomes of progressors to islet autoimmunity or T1D contained notably higher numbers of genes involved in fermentation pathways and production of Short Chain Fatty Acids (SCFA) by-products. This is relevant because some SCFA products, like butyrate, are involved in the mechanisms of gut epithelial integrity maintenance, promoting anti-inflammatory responses, and regulating the activity of regulatory T cells (58, 59). Investigating the role of the microbiome may provide insights into developing safe strategies to modulate immune regulation in infants and children.

### Secondary Prevention

Strategies for secondary prevention apply to individuals with multiple autoantibodies (at least two), with or without evidence of beta cell dysfunction. Islet autoantibodies currently represent a relevant approach in the prediction of clinical T1D. The number of autoantibodies, the age of onset, and the combination of these could be highly predictive of the progression to clinical T1D.

Recent evidence remarks how post translational modifications (PTM) of self-antigens as oxidation (60, 61), glycosylation (60), citrullination (62, 63), and deamination (64) supply neoepitopes that are able to breach immune tolerance in T1D. Strollo et al. demonstrated a new autoantibody in most of T1D individuals (61) or prediabetic children (65). They also demonstrated that the best sensitivity and specificity of the humoral biomarkers are defined by the positivity of oxPTM-INS-Ab and IA-2A, in contrast to GADA and IAA that show a lower sensitivity and specificity. In detail, the sensitivity of oxPTM-INS-Ab, IA-2A, GADA, and IAA was about 74, 71, 65, and 50%, respectively, while the specificity was 91, 91, 66, and 68%, respectively (66). They found that in GADA^+^ individuals, the further positivity of IA-2A and oxPTM-INS-Ab was the better and the more accurate combination when compared to IA-2A^+^/IAA^+^ or oxPTM-INS-Ab^+^/IAA^+^. In children oxPTM-INS-Ab^+^,GADA^+^, and IA-2A^+^ had twice the risk of progression to clinical diabetes within 5 years when compared with children with IAA^+^, GADA^+^, IA-2A^+^. At 10 years of follow-up, diabetes risk increased to 100% in the first group, compared to 84.37% in the second group (66). Although this study demonstrates the greater accuracy of oxPTM-INS-Ab in identifying progressors to T1D compared to IAA, additional studies are necessary to confirm the predictive value of oxPTM- INS-Ab in T1D.

In addition, metabolic markers have been proposed for secondary prevention. Continuous glucose monitoring (CGM) seems to have a role in predicting T1D onset in at-risk persons. Steck et al. enrolled 23 participants with positive autoantibodies who wore a CGM; they demonstrated that those children reporting a 18% or greater CGM time spent at >140 mg/dL are at increased risk to progress to clinical diabetes (67). However, to date, larger studies are needed to confirm the predictive value of CGM. Also, mild fasting or after glucose load dysglycemia increase the risk of T1D. Metabolic markers derived from oral glucose tolerance test (OGTT) accurately predict the progression to T1D in high-risk individuals (68, 69). OGTT examines the response to an artificial sugar load, CGM does not—this is the relevant advantage of this method.

Several immune interventions have been reported to delay the decline in beta-cell function (70). A promising drug is teplizumab an Fc receptor-non-binding anti-CD3 monoclonal antibody. In a phase-2 trial, Herold et al. have demonstrated that teplizumab significantly delays (by 2 years) the clinical onset of T1D in high-risk, non-diabetic relatives of diabetic patients and with at least two autoantibodies and abnormal OGTT at trial entry (71). The presence of HLA-DR4 and the absence of HLA-DR3 and of anti-ZnT8 antibodies identified the persons most likely to have a response (71). Preclinical studies suggested that an anti-CD3 monoclonal antibody needs an active autoimmune response; thus, the administration of these drugs during stage 1 of diabetes could be ineffective (72, 73).

### Tertiary Prevention

Strategies to preserve beta cell mass and/or to prolong the remission phase after T1D onset are of relevant importance, because beta cell mass rapidly declines during the first 1–2 years or following the onset of T1D; these strategies could also allow us to avoid or delay the complications of T1D (74, 75). In order to understand the immune mechanisms underlying the destruction of beta cell mass, it is key to try to halt autoimmunity and to preserve beta cell mass with the hope of eventually curing T1D. Previous pilot, randomized, placebo-controlled, single-masked clinical trial was performed with the aim to characterize the tertiary prevention strategies. Results from this study have shown that anti-thymocyte globulin ATG given at low dose (2.5 mg/kg) combined with the administration of 6 mg subcutaneously every 2 weeks for six doses of pegylated granulocyte colony-stimulating factor GCSF in individuals with T1D (duration 4–24 months) is able to preserve C-peptide (76, 77), contrary to higher doses of ATG (6.5 mg/kg) in monotherapy (78, 79). Flow cytometry analysis showed that the combination of low-dose ATG/GCSF increased the proportion of Tregs to conventional CD4^+^ T cells, while higher-dose ATG decreased Tregs proportionally (77–79). The National Institute of Health Type 1 Diabetes TrialNet Study Group (TrialNet) performed a three-arm randomized, double-masked, placebo-controlled trial (low-dose ATG/GCSF, low-dose ATG, and placebo) to compare the power of low-dose ATG/GCSF and low-dose ATG alone in preserving beta cell mass (80). This study showed that the addition of GCSF may decrease the benefits of low-dose ATG alone in the reduction of HbA1c, preservation of beta cell function, and favorable changes in immune cells subsets (80).

Many other immunotherapeutic approaches are being studied and proposed to prevent T1D. Jacobsen et al. reviewed and summarized recent interventional approaches (81), defining their proposed mechanism. Treatments include cyclosporine plus methotrexate (82), rituximab (anti-CD20) (83, 84), teplizumab (anti-CD3) (85, 86), otelixizumab (chimeric anti-CD-3) (87–89), ATG (78, 79), ATG+G-CSF (76, 77, 90), abatacept (CTLA-4/Fc fusion protein) (91, 92), *ex-vivo*-expanded autologous CD4^+^CD127^lo/−^CD25^+^polyTregs (93), autologous hematopoietic stem cell transplant (AHSCT) (94), alefacept (LFA-3/Fc fusion protein) (95), alpha-1-antitrypsin (acute phase reactant) (96, 97), canakinumab (anti-IL-1 mAb) and anakinra (IL-1-R antagonist) (98, 99), proleukin (IL2) (100, 101), etanercept (anti-TNF-α) (102), sitagliptin+lansoprazole (DPP-4 inhibitor + PPI) (103), and verapamil (104). Results of these studies are relevant in possibly offering new and promising approaches for the cure of the disease in the near future.

Vitamin D supplementation is another strategy proposed to slow the progression of the disease. In this regard, it is an ongoing randomized, placebo-controlled clinical trial to check vitamin D effectiveness in prolonging the duration of partial clinical remission (PCR), or “honeymoon phase,” increasing residual beta cell function. It began on October 19, 2017 and will conclude on July 31, 2020 (105).

In the field of tertiary prevention, it is crucial to note that about 50% of T1D patients fail to undergo partial clinical remission (106). These children, also called “non-remitters” have a prognostic disadvantage for the short- and long-term complications of T1D (107–110). A predictive model evaluating of bicarbonate <15 mg/dL, age <5 years, female sex, and >3 diabetes-associated autoantibodies has a 73% predictive power in identifying non-remission in children and adolescents with new-onset T1D (111). It is a challenge for scientists to identify this group of patients at high risk in order to properly treat them with other strategies to have a better glycemic control and to avoid or delay vascular complications.

## Conclusions

T1D is a T cell-mediated autoimmune disease characterized by selective destruction of pancreatic beta cells. The pathogenesis of T1D is very complex, and the network of factors involved needs to be better described. To date, the genetic factors are surely relevant to estimate the risk of developing T1D. In fact, the familial aggregation of T1D certainly remarks an inheritable genetic predisposition for the development of this chronic disease. Risk of T1D progression is conferred by specific HLA DR/DQ alleles (i.e., DR3/DQ2 or DR4/DQ8), but it is important to note there are also alleles that would seem to be protective factors for the development of T1D (i.e., DQB1^*^0602).

In addition, non-HLA genes are also involved in the polygenic inheritance of T1D.

Although, the genetic factors certainly have an important role in the risk of T1D, the concordance rate not equal to 100% between monozygotic twins underlines the importance of possible environmental factors and the crucial aim to define them to truly predict and prevent T1D. Among the potential factors related to the risk of progression to T1D, the positivity of multiple autoantibodies is demonstrated to be a major risk factor of developing insulin-requiring diabetes. The role of infections, diet, and other still unknown factors potentially involved in the pathogenesis of T1D have to be better investigated to accurately predict the risk of T1D. These studies will pave the way to studies for primary and secondary prevention of the disease, with the final aim of avoiding or limiting insulin-dependence. Finally, strategies of tertiary prevention are necessary to delay or prevent diabetes-related complications.

## Author Contributions

FC and CG reviewed the paper. MP wrote the manuscript.

## Conflict of Interest

The authors declare that the research was conducted in the absence of any commercial or financial relationships that could be construed as a potential conflict of interest.
